# The State of Public Health Lead Policies: Implications for Urban Health Inequities and Recommendations for Health Equity

**DOI:** 10.3390/ijerph16061064

**Published:** 2019-03-24

**Authors:** Alana M. W. LeBrón, Ivy R. Torres, Enrique Valencia, Miriam López Dominguez, Deyaneira Guadalupe Garcia-Sanchez, Michael D. Logue, Jun Wu

**Affiliations:** 1 Department of Population Health & Disease Prevention, Program in Public Health, Susan and Henry Samueli College of Health Sciences, University of California, Irvine, CA 92697, USA; irtorres@uci.edu (I.R.T.); mdlogue@uci.edu (M.D.L.); junwu@uci.edu (J.W.); 2 Department of Chicano/Latino Studies, School of Social Sciences, University of California, Irvine, CA 92697, USA; 3 Orange County Environmental Justice, Santa Ana, CA 92705, USA; enrique@ocej.org; 4 Jovenes Cultivando Cambios, Santa Ana, CA 92705, USA; miriamlopez123490@gmail.com (M.L.D.); deyaneira.garcia.sanchez@gmail.com (D.G.G.-S.); 5 Center for Occupational and Environmental Health, School of Medicine, University of California, Irvine, CA 92617, USA

**Keywords:** lead, lead prevention policies, lead paint, soil lead, lead in water, health disparities, health inequities, environmental justice, environmental racism

## Abstract

Although lead has been removed from paint and gasoline sold in the U.S., lead exposures persist, with communities of color and residents in urban and low-income areas at greatest risk for exposure. The persistence of and inequities in lead exposures raise questions about the scope and implementation of policies that address lead as a public health concern. To understand the multi-level nature of lead policies, this paper and case study reviews lead policies at the national level, for the state of California, and for Santa Ana, CA, a dense urban city in Southern California. Through a community-academic partnership process, this analysis examines lead exposure pathways represented, the level of intervention (e.g., prevention, remediation), and whether policies address health inequities. Results indicate that most national and state policies focus on establishing hazardous lead exposure levels in settings and consumer products, disclosing lead hazards, and remediating lead paint. Several policies focus on mitigating exposures rather than primary prevention. The persistence of lead exposures indicates the need to identify sustainable solutions to prevent lead exposures in the first place. We close with recommendations to reduce lead exposures across the life course, consider multiple lead exposure pathways, and reduce and eliminate health inequities related to lead.

## 1. Introduction

In recent decades, there has been increasing attention amongst communities, environmental agencies, and policymakers to disparate exposures to environmental hazards that have traditionally differentially affected communities of color and low-income communities [1,2]. The water crisis in Flint, MI, USA has heightened attention to environmental racism and in particular synergistic racial and socioeconomic inequities in lead exposures and their social, economic, and health consequences [3]. The experience of residents in Flint points to the persistence of exposure to lead, the importance of recognizing and addressing lead exposures from multiple sources, the continuing existence of inequities in lead exposures, and the struggle for governmental agencies to actively listen and respond to resident concerns about environmental injustices. The water crisis in Flint surfaced critical questions about federal, state, and local public health policies related to preventing environmental lead exposures, addressing existing lead exposures, and promoting health equity. 

Evidence linking exposure to lead—a neurotoxin—with adverse health outcomes has yielded policies to prevent the sale of lead-based paint and leaded gasoline to the general public in the U.S. and has contributed to bans on lead plumbing and lead solder in canned goods in the U.S. [4]. Despite this progress, Americans continue to be exposed to lead through several pathways. These exposure pathways include lead-based paint in older homes [5,6]; lead-contaminated water distribution systems [3,7]; lead in dust and soil, which may have deposited through mechanisms such as lead-based paint from older houses, soil contaminated by traffic exhaust from historical leaded gasoline, or other industrial emissions [5,8,9,10,11]; and/or industrial or smelter emissions [12,13]. Lead has also been identified in urban agricultural settings [8,14]. Notably, residents of urban areas, residents of color, and low-income residents are at greatest risk for lead exposures [5,13,15,16]. Working in industries that confront historical and ongoing uses of lead in products, such as lead industries (e.g., radiator repair, lead processing); electronic waste recycling; and construction, especially demolition work, also poses risk of lead exposures for workers and their households [17,18,19,20,21,22,23]. These hazardous industries disproportionately employ groups who have been historically disenfranchised [24]. In consequence, take-home lead exposures from working in these industries may compound lead exposure pathways already present in their community [24]. The persistence of and inequities in lead exposures raise questions about the scope and implementation of policies that address lead as a public health concern, with implications for individual, family, and community health.

Since 2017, our community-academic partnership has been working to understand and address lead exposures in a predominantly Latina/Latino/Latinx community in Santa Ana, California, USA. Our partnership process has surfaced important questions about the roles and responsibilities of governmental entities who construct and implement lead-related policies, and/or respond to lead exposures. For example: *What policies hold industries accountable for lead contamination? What policies prevent lead in consumer products and the environment? If a child is found to have high blood lead levels but no action has been taken to reduce lead exposures, what governmental agency is charged with ensuring that public health, health care, and educational systems work together to mitigate and eliminate lead exposures and reduce harm? Are there similar protections for adults with high blood lead levels? How do governmental agencies incorporate scientific evidence to address lead exposures and enforce lead remediation policies? How do residents navigate the systems responsible for mediating or addressing lead exposures? What are the points of entry that activate lead mitigation and remediation? How, if at all, do these policies apply a health inequities framework?*

Community-academic partnerships are well-positioned to close the gap between community knowledge, scientific evidence, and policies that are needed to promote health and health equity [25,26]. Several longstanding community-academic partnerships are working to understand and address the health impacts of environmental injustices [26,27,28,29,30]. However, fewer partnerships have focused on the health implications of lead exposures across the life course, sources of lead in the community, and the translation of community and scientific knowledge about lead into multi-level policy and programmatic interventions. Notably, environmental policies are often constructed absent of the perspectives of communities who are most affected by these policies. Additionally, lead-related policies have long trailed the robust scientific evidence indicating that no level of lead is safe for human health [3,4]. The persistence and continuing emergence of lead as a health and health equity concern points to the shortcomings of existing policies to prevent or address lead exposures across the life span and their implications for health inequities. Accordingly, it is critical to revisit existing lead-related policies through a community-academic partnership lens to bring together multiple forms of knowledge and evidence to inform equity-oriented solutions regarding the development, implementation, and assessment of lead policies.

Through a community-academic policy assessment process, this paper provides a critical review of lead policies at the national level, for the state of California, and for the city of Santa Ana, CA, a dense urban area in Southern California. By focusing on lead policies across three jurisdictions, this review serves to enhance understanding of how institutions charged with promoting the public’s health are currently addressing lead as a public health concern. It focuses on implications for health inequities, a term that refers to differences in health status that are preventable, systematic, unjust, and actionable [31]. Thus, this review seeks to inform public health policy and action to prevent lead exposures and health inequities linked with environmental injustices. In particular, this collaborative synthesis of policies can serve as a tool for partnerships confronting similar questions. 

### 1.1. Lead Exposures: Implications for Health and Health Inequities Across the Life Course

Children are particularly vulnerable to the health impacts of environmental lead due to higher absorption levels, developmentally appropriate hand-to-mouth activity, and child neurocognitive development. Despite strong scientific evidence that no level of lead is safe for young children [32], children continue to be exposed to lead, with implications for cognitive, academic, carceral, and health outcomes throughout the life course. Evidence links early life lead exposure with adverse neurological and cognitive outcomes, such as reduced brain volume, diminished IQ, lower working memory and processing speed, and reductions in perceptual reasoning [33,34,35,36,37]; poor school performance [38]; asthma [39,40,41,42]; and incarceration [43,44]. One study, in particular, found that reducing children’s blood lead levels to zero decreased the probability of scoring below proficiency in reading and math by 22% and 13%, respectively [45]. 

Blood lead levels are commonly used to measure lead exposure especially in children due to the convenience and the relatively low cost in testing. However, blood lead may not be the best indicator of the full risk for lead-associated toxicity. This is because blood lead has a median biologic half-life of about 1 month, thus mostly reflects recent or current lead exposure levels, while bone lead has a half-life of 20–30 years which better reflects chronic or cumulative exposure levels [46]. In addition, skeletal bone has been found to comprise 90–95% of lead burden in adults and 80–95% in children [47,48]. Accordingly, storage of lead in the body is a dynamic process and is different in blood (short-term) and bone (long-term) [49].

Later in the life course, lead exposures are associated with cardiovascular risk, renal problems, and adverse cognitive outcomes for older adults [34,50,51,52,53]. These health problems are exacerbated among older adults with osteoporosis, whom experience the release of lead accumulated in their bones into their bloodstream [52]. As women are more likely to experience osteoporosis than men [54], this literature suggests that osteoporosis is one mechanism by which lead exposures disparately burden older women. Moreover, relatively low blood lead levels in adults are associated with elevated risk of death from all-causes, cardiovascular disease, and cancer [55,56]. Indeed, one study attributed 412,000 deaths annually to low-level environmental lead exposures among adults in the USA [56]. 

The literature reviewed above focuses on the health impacts of lead on an individual level, with implications for population health and health inequities. Moving to a family and community health perspective, evidence indicates two key pathways by which lead exposures affect households and multiple generations. First, lead exposures among adults—such as occupational lead exposures—may be transferred to other household members, including children [22,57]. Second, during pregnancy blood lead freely crosses the placenta and is absorbed by the fetus during gestation [57,58]. Gestational exposures to lead may reflect current lead exposures, or lead stores in bones accumulated over the life course. Thus, child blood lead levels—the most commonly available indicator of lead exposures—likely reflect exposure pathways for older children, youth, and adults in a given household or community. Together, these literatures indicate that failing to protect individuals of all ages from lead exposure contributes to debilitating consequences across the life course and across generations. 

Nationally, blood lead levels have decreased across the United States [15,59,60], due in part to the removal of lead-based paint and leaded gasoline from the U.S. For example, from 1999–2002 to 2007–2010, the percent of children one to five years of age with blood lead levels ≥5 µg/dL decreased from 8.6% to 2.6%—representing a 70% decline over this period [60]. Among adults, the proportion of adults with blood lead levels ≥10 µg/dL decreased from 3.3% in 1988–1994 to 0.7% in 1999–2002 [59]. Despite reductions in the percent of children and adults across the U.S. with elevated blood lead levels, a robust evidence base indicates that there is no safe blood lead level [60]. 

Additionally, lead exposures—commonly assessed by child blood lead levels—are not evenly distributed across the U.S. population. In 2012, the Centers for Disease Control and Prevention classified a reference level of 5 micrograms per deciliter (µg/dL) to indicate children with elevated blood lead levels [32,61]. Black [15] and Latina/o/x children [15,16] are at greatest risk for high blood lead levels, as are residents of low-income households [15]. Risk factors for residence-based lead exposures include living in: older homes [15], lead-contaminated homes [6,62,63], urban areas [11,13], residences situated along high-traffic routes such as freeways or high-traffic arterial roads [11,13]; residences adjacent to metal mining and smelting [64]; and living with family members exposed to lead in the workplace [19,20,65]. Additionally, the health consequences of lead exposures are exacerbated under stressful life conditions [66], which are disparately experienced by communities of color and low-income communities [2,67,68,69]. 

These persistent inequities point to shortcomings of existing lead policies and practices. Addressing these inequities requires policies and practices focused on preventing lead exposures, remediating the source(s) of lead, and mitigating the health consequences of lead exposures, with a focus on promoting health equity. 

### 1.2. Summary of Findings

This policy review includes an examination of lead exposure pathways (e.g., paint, soil, dust, and/or water) addressed by lead policies; the level of intervention (e.g., prevention, mitigation, remediation); and how, if at all, health inequities are incorporated into lead policies. We find that lead policies largely focus on establishing hazardous lead exposure levels in settings and consumer products, disclosing lead hazards, and remediating lead paint. Several policies focus on mitigating exposures once children have been exposed to lead (as indicated by elevated child blood lead levels), rather than prevention. In light of notable gaps in existing policies, we conclude by discussing recommendations to prevent lead exposures and to promote health equity. 

## 2. Materials and Methods 

### 2.1. Community-Academic Partnership

The (Anti-)Soil Lead Project is a community-academic partnership that has been working together since 2017 to understand and address soil lead exposures in Santa Ana, CA, a dense urban area in northern Orange County. Santa Ana is a predominantly Latina/o/x and immigrant community [70,71] (Table 1), and children in Santa Ana are 64% more likely to have elevated blood lead levels relative to children across California [72]. Our partnership, which includes Orange County Environmental Justice (OCEJ), Jovenes Cultivando Cambios (JCC; Youth Cultivating Changes in English), and academic partners at the University of California, Irvine (UCI), seeks to understand soil lead exposures for residents and to identify opportunities to prevent lead exposures linked with legacy lead and/or ongoing sources of lead. Our partnership was spawned by a joint interest in addressing environmental injustices in the region, as well as concern about high levels of soil lead identified in some Santa Ana neighborhoods by Cabrera [73], an investigative reporter. Cabrera [73] reported that several areas in Santa Ana had soil lead levels three to ten times higher than Environmental Protection Agency cut-off points for lead toxicity (400 parts per million (ppm)) [74]. Following publication of the report, in summer 2017, Cabrera discussed findings with several community-based initiatives and neighborhood organizations working to address tenant rights, gentrification, food sovereignty, and reform of criminal justice and immigrant policing systems. Linked with the strong community organizing history in Santa Ana, community concerns about lead exposures have remained salient and have been connected to several local social movements. These discussions activated questions including: *What is the distribution of soil lead across Santa Ana? Who is at greater risk for exposure to soil lead? What are the sources of soil lead in Santa Ana? Why are children in Santa Ana more likely than children across California to have elevated blood lead levels? What are policy solutions to mitigate and remediate soil lead? What actions can residents take to protect their wellbeing and to advocate for change?* Following a series of formal and informal community conversations, OCEJ and JCC, an emerging environmental justice organization and youth cooperative working towards food sovereignty, respectively, identified a need to further examine soil lead levels in Santa Ana. OCEJ and JCC in turn invited academic partners at UCI with whom there were longstanding and emerging relationships to participate in this planning process. This process culminated in a follow-up study of soil lead levels across the city of Santa Ana, which is currently underway. The (Anti-)Soil Lead Partnership is informed by local community knowledge about environmental concerns, as well as the wisdom of other communities (e.g., Los Angeles, San Diego) working to address environmental injustices. Building upon this knowledge, we are continuing to elicit the perspectives and priorities of residents through multiple community engagement strategies, including surveys of adult and youth residents and past and ongoing community forums and dialogues. Our partnership also identified the need to review and analyze lead-related policies in order to identify policy-oriented solutions to prevent, mitigate, and remediate lead exposures. 

Informed by a shared understanding that youth are inheriting a world that is characterized by multiple environmental injustices, youth organizing has been central to our partnership process. The leadership, wisdom, social imagination, and visions of young people are urgently needed to heal and protect the environment and communities who have been disparately affected by environmental injustices. JCC youth leaders have highlighted questions about the connections of lead exposures to educational outcomes and behaviors that are vulnerable to punishment and policing in the school system in Santa Ana, CA and across California; the criminalization of communities of color who have been affected by environmental injustices; and implications of lead exposures for health across the life span [81]. Likewise, as residents navigate, ameliorate, and resist gentrification processes unfolding in Santa Ana, concerns about lead exposures—and in particular soil lead—have intersected with housing justice and food sovereignty movements locally. These local social movements reflect the interconnections between environment, education, housing, food, carceral, and health-related concerns. Building on this youth organizing model and histories of activism in Santa Ana [82], our partnership seeks to build an environmental justice movement in Santa Ana, CA that thoughtfully engages youth, adults and elders, with equity, healing, and regeneration at the center of our process and vision. 

### 2.2. Policy Review & Analysis

We identified policies at the federal, state (California), and local (Orange County, CA; City of Santa Ana, CA; Santa Ana Unified School District) level that address lead prevention, exposures, and/or mitigation. Federal lead-related policies were identified using the Environmental Protection Agency (EPA) website. Laws and executive orders listed under the laws and regulations section of the EPA’s lead webpage were reviewed. California policies were identified using the Childhood Lead Prevention Branch webpage located on the California Department of Public Health website. The decision to use federal and state agency websites to identify lead policies was made to reflect the process communities would undertake to obtain information on lead policies. Specifically, a first step in identifying lead policies involves visiting websites of federal and state agencies tasked with protecting human and environmental health. It should be noted that policies not included in these websites were not reviewed. When considering local lead-related policies, we identified the need to review policies enacted by the local health department and City of Santa Ana, CA. Additionally, youth raised important questions about school district policies pertaining to lead exposures in school and/or programs and policies to mitigate the effects of lead on educational outcomes. Thus, our review of local policies included three jurisdictions: Orange County Healthcare Agency (local health department); City of Santa Ana, CA; and the Santa Ana Unified School District. Two reviewers (first and second author) reviewed the policies to identify the level of intervention addressed (e.g., prevention of lead exposures; notification of lead levels in consumer products or removal of lead from setting; lead exposure mitigation—including case management; and remediating settings contaminated with lead); whether policies addressed health inequities, legacy lead exposures, or current or future lead exposures; the source of lead (e.g., water, paint, soil, consumer products); the setting of potential lead prevention or remediation intervention (e.g., residential, commercial) and implications for health inequities. Co-authors reviewed and discussed this analysis, and identified recommendations for strengthening policies to reduce lead exposures, attend to lead exposures from multiple sources, and address health inequities linked with lead. 

## 3. Results

Presented in Table 1 is an overview of census-based features of the physical environment and social and economic characteristics of residents in the regions of focus for this policy assessment: the United States; state of California; Orange County, CA; and the City of Santa Ana, CA. According to 2010 Census estimates, nearly all residents of the City of Santa Ana (100.0%) and Orange County (99.9%), CA live in areas designated as urban (defined as a minimum population density of 500 persons per square mile), slightly more than the average across California (95.0%) and greater than patterns across the United States (80.7%). While more than half of the housing stock across the United States (54.7%) was constructed before 1980, approximately six in ten houses across California (60.2%) and Orange County (61.6%) were built before 1980. By stark contrast, approximately eight in ten (81.1%) houses in Santa Ana were built before 1980, shortly after lead was banned from paint sold in the U.S. Thus, the majority of housing stock across Santa Ana may have had or may still contain lead-based paint, pointing to the intersection of environmental and housing justice concerns in Santa Ana, CA. 

In 2013–2017, though one in ten Orange County households (10.7%) had incomes below the federal poverty level, more than one in six Santa Ana households (17.6%) met these criteria. The population across Santa Ana was also younger than patterns across the nation, state of California, and Orange County during this period: More than one-third of Santa Ana residents (35.1%) were 18 years of age or younger, compared to approximately 30% across the U.S., California, and Orange County. Likewise, in 2013–2017 the majority of residents across Santa Ana, CA identified as Latina/o/x (77.3%) and nearly half were born outside of the U.S. (45.0%). Moreover, 78.9% of residents in Santa Ana reported having health insurance over this period, ten percentage points below the average across the U.S., California, and Orange County. 

For each level of policy intervention (federal, state of California, Orange County/Santa Ana, CA), this case study begins with a brief overview of lead-related policies and examines gaps in these policies, with a focus on implications for persistent and emerging health inequities. Federal policies are organized chronologically to illustrate the evolution of lead-related policies, while California state policies are organized thematically. This structure emerged from themes that surfaced in the policy analysis, suggesting federal policies issue guidelines and mandates for lead abatement and remediation activities and California state policies amplify federal policies, fill gaps, and address unique conditions in the state. Similar to state policies, local policies are organized thematically, reflecting local implementation of state-level policies and/or development of local policies to address local conditions within their jurisdiction. Our partnership process identified the importance of examining policies at the school district level, particularly with respect to questions of how school districts implement or augment federal and state policies regarding levels of lead in areas where children are exposed. While our review did not identify school district policies that explicitly pertained to lead, we have retained this review in the Results section and highlighted gaps in these policies. The discussion section in turn weaves together this multi-level patchwork of lead-related policies to identify gaps across the social-ecological spectrum and enumerate implications for health inequities. 

### 3.1. Federal Lead Policies

As shown in Table 2, federal lead policies largely focus on establishing safe and unsafe levels of lead exposure, which in turn inform policies regarding lead remediation activities. 

Federal policies primarily focus on remediating lead-based paint hazards, water, and sites that have received official designation as toxic from the Environmental Protection Agency (EPA). The following section describes federal policies that explicitly address lead exposures and includes an assessment of implications for health equity.

#### 3.1.1. Clean Air Act

The Clean Air Act (CAA) was passed by Congress in 1963 and amended in 1970 and 1990; its purpose is to reduce air pollution [83]. Under the CAA, lead is defined as a criteria pollutant, along with particulate matter, ground-level ozone, carbon monoxide, sulfur oxides, and nitrogen oxides. As a result of the CAA, leaded gasoline began to be phased out after the 1970 amendment and was finally banned in 1996, following the 1990 amendment to the policy. It should be noted that CAA regulations do not currently apply to emissions from aircrafts that use leaded gasoline [84].

The banning of leaded gasoline dramatically improved blood lead levels among people of all ages and has significantly reduced lead in the air [85]. Yet, the CAA falls short in protecting communities from lead exposure. First, the policy does not address remediation strategies for land contaminated by historical use of leaded gasoline, which continues to have real health consequences for children, youth, and adults today [11,13]. Second, the CAA does not regulate aviation gasoline. Communities located near airports, such as Santa Ana, CA, are made vulnerable to lead emissions released by aircrafts (often small aircrafts) that use leaded gasoline. The lack of regulations results in lead emissions that often exceed the National Ambient Air Quality Standards for lead [86]. Consequently, leaded aviation gasoline has been found to be associated with increased blood lead levels among children living near airports [87] and among aircraft maintenance crewmembers [88].

#### 3.1.2. Safe Drinking Water Act and Amendments

The Safe Drinking Water Act (SDWA) of 1974 seeks to improve the country’s water system for safe, public consumption and applies to all public water systems [89]. The lead-specific guidelines put forth by the SDWA prohibit the use of leaded pipes in any public water system and within any plumbing located in residential or non-residential facilities that provide water for human consumption [89]. Under the SDWA, in the event of lead contamination in drinking water due to the materials of the water distribution system and/or lead leaching due to corrosivity, the owner and/or operator of a public water system must notify all individuals affected. The notice must include information on the source of lead, potential health consequences, methods for mitigating lead, steps the water system is taking to remediate the situation, and the need to seek alternative water sources [89]. In 1988, Congress amended the SDWA via the passage of the Lead Contamination Control Act (LCCA) [90]. The policy instructs the EPA to classify drinking water coolers with lead-lined tanks as hazardous. Further, the policy instructs the EPA to create and distribute a list of brands and models of lead-based water coolers and a testing protocol for schools to test their water and ways to remediate the contamination. Under the LCCA, states are required to distribute the EPA’s list and testing protocols to educational institutions that serve children under the age of six. In addition, the LCCA establishes a grant program to expand lead screening efforts among young children, interventions for lead-poisoned children, and educate the public about childhood lead poisoning in areas considered high-risk [90].

The purpose of the SDWA is to increase the safety of water consumed by the public, however, the policy may be falling short when it comes to lead. As defined by the SDWA, lead-free pipes, solder, and flux can contain lead. Specifically, solder and flux can contain 0.2% lead, pipes and pipe fittings can contain up to 8% lead, and plumbing fittings and fixtures can consist of 4% lead [89]. However, as has been noted in the literature, there are no safe levels of lead [36,37,45,91]. Trace levels of lead that might leach from pipes, solder, and flux that meet the SDWA definition of lead-free are not the sole source of lead to which children and adults are exposed. These trace amounts can compound other pathways of lead exposure—paint, soil, and dust—and elevate children’s blood lead levels. Further, lead exposure in the United States is highly racialized [92,93], and compounding lead exposures that result from such lax definitions are more likely to negatively impact children of color. Until the SDWA calls for the complete elimination of lead in the pipes that distribute water for public consumption—and removes lead from existing water distribution systems—assurances of safety of water from lead will not be met. 

#### 3.1.3. Toxic Substances Control Act and Related Policies

The Toxic Substances Control Act (TSCA) was passed by Congress in 1976 to regulate new chemicals and chemical mixtures that pose a significant health risk and to provide guidance on the reduction and abatement of existing toxic substances including lead [94]. Since 1976, subsequent policies have been passed to fulfill the requirements of the TSCA. These policies are described below: 

##### The Residential Lead-Based Paint Hazard Reduction Act (Title X) 

Title X was passed in 1992 and requires the disclosure of known lead-based paint and lead-based paint hazards in housing units built prior to 1978 from landlord to tenant, or from seller to purchaser [95]. Sellers and landlords are required to make available any lead hazard evaluation reports conducted in the housing unit of interest, including reports on common areas and units not of interest in apartment buildings. Title X grants the purchaser and prospective tenant a 10-day period during which they can hire a third-party organization to conduct a risk assessment. Prior to finalizing the sale of a home built prior to 1978, the seller must provide the buyer with a lead warning statement, which they must sign. Notably, federal funding is available to states or local governments to test soil, dust, and child blood lead levels (BLL) where lead reduction activities occurred, with the exception of federally assisted housing, federally owned housing, and public housing. Finally, Title X outlines the guidelines for evaluating, reducing, and notifying tenants of lead-based paint hazards in federally owned residential property or in homes receiving federal assistance [95].

##### Hazard Standards for Lead in Paint, Dust and Soil (Standards) 

Passed in 2001, the Standards set standards for lead-based paint hazards—lead-based paint, dust, and soil—in pre-1978 homes and in facilities in which children under the age of six spend a significant portion of their day (e.g., daycare centers). The Standards identify lead as hazardous when lead in dust is equal to or greater than 40 µg/ft^2^ on floors and 250 µg/ft^2^ on interior window sills. Lead in soil is classified as hazardous when lead levels are greater than or equal to 400 ppm in soil in children’s play areas or an average of 1200 ppm for soil in the yard (not including children’s play area) [96]. Chipping paint on contact surfaces and on chewable surfaces are also identified as hazardous. It is important to note that these Standards do not mandate action if lead levels are below the standards identified above [96].

##### Lead Renovation, Repair and Painting Program Rules (RRP) 

Established by the EPA in 2008, the RRP addresses construction-related activities that would disturb lead-based paint hazards [97]. Under the RRP, workers are required to be certified and receive training on safe practices when working with lead-based paint hazards. In addition, the RRP requires organizations that engage in lead-related construction activities to be certified by the EPA [97].

##### Consolidated Enforcement Response and Penalty Policy for the Pre-Renovation Education Rule; Renovation, Repair and Painting Rule; Lead-Based Paint Activities Rule (Penalty Policy)

While the TSCA sets penalty maximums for individuals that violate this policy, the Penalty Policy provides guidance to the EPA on appropriate enforcement response and quantifies penalty amounts for violations [98].

Taken together, these policies maintain a narrow scope that falls short in protecting communities at greatest risk for lead exposure. This is evident in the TSCA’s mandate that regulation not impede or “create unnecessary economic barriers to technological innovation” [94]. This policy framing privileges industry over people while simultaneously limiting protections for communities that might be disproportionately exposed to harmful substances, including lead. Notably, Title X places an unfair burden on the renter or homeowner to hire someone to conduct this assessment without any financial assistance and within a ten-day period, and to make decisions about their potential living arrangements within a short time period and a potentially volatile housing market. Additionally, in the event that lead is found in the home, it is unclear that the prospective homebuyer or tenant will receive federal assistance in remediating the lead-based hazards in the home if no child under the age of six will live there. Further, in considering a range of housing arrangements, Title X surfaces questions about which sub-populations need but do not receive federal assistance in remediating lead exposures. For example, residents with informal leases, unauthorized/undocumented legal statuses, and/or felony convictions may be uncertain about both their housing rights and rights related to assessing and addressing lead in the home [99]. The Standards continue to focus on lead-based paint, as they are only applicable to homes built pre-1978, the year during which lead-based paint was outlawed. In doing so, the Standards overlook other historical sources of lead, such as lead from gasoline emissions, which was not completely phased out until the mid-1990s [85]; and lead in soil and dust that may be influenced by leaded paint. 

As with Santa Ana, CA, residents of color and low-income households tend to live in homes constructed before 1978 and in areas characterized by lead in soil and dust [6,62,63]. TSCA and related lead prevention and remediation policies that do not attend to the inequitable concentration of lead exposures in residential areas serve to preserve and exacerbate lead-related health inequities. 

#### 3.1.4. Comprehensive Environmental Response, Compensation, and Liability Act

In 1980, Congress passed the Comprehensive Environmental Response, Compensation, and Liability Act (CERCLA), also known as Superfund. Under CERCLA, chemical and petroleum companies are taxed to create a fund for cleaning abandoned or uncontrolled hazardous waste sites [100]. The policy grants the EPA the authority to obtain the cooperation of responsible parties and recover costs for the cleanup. However, when a responsible party cannot be identified, the EPA is charged with paying for the clean-up. It should be noted that CERCLA authorizes two forms of responses: short-term removals (e.g., event that requires immediate action) and long-term responses (e.g., a site that does not pose an immediate threat but contains hazardous substances) [100]. Lead is identified as a common contaminant at superfund sites by the EPA, indicating the prevalence of lead hazards in the environment [100].

This policy supposes a robust EPA structure committed to assessing and remediating contaminated sites and holding responsible parties accountable. This policy privileges the conceptualization of superfund or toxic sites as specific to locations in which an industry is or was located. Missing from this policy is a consideration of broader environmental and community-level impacts, such as the runoff of pollutants into a watershed and the suspension of contaminants into the air and soil, and further to residences, and businesses nearby. Given that toxic sites are largely concentrated in communities of color [1,101,102], attention to community-level impacts and exposure remediation across a broader geographic spectrum is needed. It should be noted that a site can only be cleaned if it appears on the National Priorities List (NPL) [100]. While community members can notify the EPA of a hazardous site, it remains unclear if the EPA pre-screens all sites for superfund assessment, the first step in adding a site to the NPL, and the timeline between receiving notification and conducting the pre-screening. Opportunities for community involvement are identified at each step of the clean-up process once a site is selected for assessment. Community input is primarily solicited via a public comment period announced in the local media during the assessment process. The remediation phase presents more opportunities for community involvement via forming a Community Advisory Group, applying for a Technical Assistance Grant, or applying for Technical Assistance Services for Communities. The structure for community input supposes communities are aware of and organized to address a particular hazardous site and prepared to engage with the EPA. Without knowing the extent to which communities are organized, community input can become a symbolic gesture.

### 3.2. California State Policies

As is indicated in Table 3, California state policies are comprehensive in their scope. They amplify lead policies enumerated by the federal government, and address gaps left in the federal lead policy framework. California’s policies address housing, occupational settings, consumer products, water, and the testing of blood lead levels in young children.

#### 3.2.1. Housing

Housing standards for human occupancy (Section 1941.1) and requirements for the disclosure of lead-based paint hazards to buyers (1102 to 1102.16) are outlined in the California Civil Code. California Health and Safety Codes allow enforcement of civil penalties in the event a child becomes lead poisoned due to the presence of lead hazards in the home (Section 17961); describe actions and proceedings on behalf of the regulating agency and the guilty party in the event of a housing violation (Section 17980); establish reporting requirements for blood lead testing laboratories (Section 124130); establish the definition of lead hazards—deteriorating lead-based paint, lead-contaminated dust, lead-contaminated soil, or disturbing lead-based paint without containment (Section 17920.10); and stipulate that only individuals certified in lead-construction activities can engage in construction activities that would disturb lead and grants local enforcement agencies with the authority to issue cease and desist notices in the event that construction activities are creating lead hazards (105251–105257) [103].

While these policies aim to improve housing conditions by limiting the presence of lead hazards in the home, it remains unclear whom is the responsible party for testing for lead hazards in rental units that are not undergoing renovation. As the codes are currently written, it appears enforcement is only applied in homes undergoing renovation and when children’s blood lead levels exceed 10 µg/dL, the current threshold at which the state requires intervention. Ten micrograms per deciliter far exceeds the Centers for Disease Control and Prevention (CDC) reference level of 5 µg/dL, the level at which the CDC recommends intervention [104]. The only way to determine if a child has exceeded the 10 µg/dL threshold is through a blood test because lead poisoning under 60 µg/dL is asymptomatic [105]. However, nearly three-fourths of young children enrolled in Medi-Cal, a public health insurance program (Medicaid) for low-income individuals and families in the state of California, go untested for lead, despite insurance mandates [106]. The passive strategy of waiting for a child to have a blood lead level above 10 µg/dL is harmful to children given evidence indicating that blood lead levels as low as 2.5 µg/dL can cause significant cognitive declines in children [45]. Notably, protections for older children, adults, and elders are completely absent in state-level housing policies.

#### 3.2.2. Occupational Settings

Sections 105185 through 105197 of the California Health and Safety Code establish an occupational lead prevention program to monitor adult lead poisoning cases, establish a registry of these cases, identify sources of lead, investigate take-home exposures, provide training to workers on lead poisoning prevention in occupational settings, and define adult lead poisoning as a blood lead level equal to or greater than 25 µg/dL. The California Health and Safety Codes establish guidelines to accredit trainers leading lead-related construction activity trainings and certify individuals to engage in lead-related construction activities (Section 105250). Title 8 of the California Code of Regulations beginning with section 1531.1 establishes lead exposure maximums for construction work where employees might be exposed to lead—50 µg/m^3^ of air average over an eight-hour period; requires the monitoring of employees, the provision of personal protective equipment (PPEs), and communication between employers and employees on the health risks of lead; and the maintenance of records including exposure assessments, medical surveillance, and medical removals [103]. 

California’s permissible lead exposure level for adults (25 µg/dL), is less stringent than the 5 µg/dL established by the National Institute of Occupational Safety and Health (NIOSH) [85]. This is notable provided lead exposure in adulthood has been found to be associated with hypertension, renal problems, cognitive deterioration, and an elevated risk for all-cause mortality [51,52,53,55]. While these codes and regulations intend to keep workers safe, evidence links construction work to elevated blood lead levels, especially among those that engage in demolition work [22,23], indicating a gap between policy and practice. The codes raise questions about their applicability in informal work arrangements. Day laborers are routinely hired for residential construction jobs, including home renovations, by homeowners and small construction firms [107,108]. These work arrangements are informal and vary on a day-to-day basis. Day laborers are rarely equipped with proper training and safety equipment [108,109,110]. One can conclude that day laborers might be engaging in lead-related construction activities without proper protective equipment. Adding to day laborers’ vulnerability is their immigration status. The majority of day laborers are immigrants from Latin America who often lack legal status in the U.S. [111]. Without legal status, immigrant day laborers are left with few protections and little to no legal recourse [112] to prevent being exposed to lead. This is heightened by the reality that small construction firms typically fall outside the purview of enforcement agencies such as Occupational Health and Safety Administration (OSHA).

#### 3.2.3. Education

The Lead-Safe Schools Protection Act (1992) called for the development and implementation of a survey to identify public day care facilities, pre-schools, and elementary schools that have lead hazards (California Education Code, Section 32241), estimate the extent of the presence of lead hazards in public daycare facilities, pre-schools, and elementary schools and develop cost-effective measures for remediation (Section 32242), mandate that schools found to have lead hazards inform parents, teachers, and staff (Section 32243), and ban the use of lead-based paint, lead-based plumbing and solders, and any other sources of lead in the construction or renovation of schools (32244). The survey collected risk-factor information from a sample of schools, including “location in relation to high-risk areas, age of the facility, likely use of lead paint in or around the facility, numbers of children enrolled under the age of six, and results of lead screening programs” [113]. The survey was implemented in 1998 [103].

Although the survey was carried out in 1998, it remains unclear if schools assessed to contain lead hazards on their premises were required to remediate the lead source. Section 32243 includes a directive to inform teachers, staff, and parents of the survey results in the event lead was found on the premises of the school within 45 days of the release of the survey results. A similar mandate is not made for lead remediation. The section notes that schools that “undertake any action to abate existing risk factor for lead shall utilize trained and state certified contractors, inspectors, and workers” [113]. This suggestion for how to remediate lead hazards does not equate to a directive mandating lead remediation. This policy does not make funds available to schools for lead abatement activities, raising questions about equitable access to a lead-free learning environment. Further, this is survey was not a statewide mandate. Instead, a sample of schools were selected to participate in the study to estimate patterns of lead hazard in schools state-wide. Schools that were not assessed may not be aware of the presence of lead hazards.

#### 3.2.4. Consumer Products

California Health and Safety Codes prohibit the sale and manufacture of children’s toys containing lead-based paints or lacquers that exceed the lead content permitted by the federal government (Sections 108550–108580), prohibit the sale of candies with lead levels exceeding the state’s definition of “naturally occurring”—“not avoidable by good agricultural, manufacturing, and procurement practices, or by other practices currently feasible” (Section 110552), and limit the amount of permissible lead and other toxic substances in jewelry to 0.02%-0.06% by weight (Sections 25214.1–25214.4.2). 

The definition of candy in Section 110552 specifically calls out tamarind and chili, which are typical ingredients in Mexican candies, suggesting only candies containing tamarind and/or chili contain lead and should be the subject of close monitoring. However, a report by the Food and Drug Administration (FDA) identified chocolate, peanuts, and raisins as potential sources of lead in candies consumed by young children [114]. Yet, this policy makes no specific reference to these ingredients. Further, in the late 1990s and early 2000s there were a handful of cases in California in which Latina/o/x children experienced blood lead levels above 20 µg/dL [115,116]. In each instance where candy was blamed for the child’s elevated blood lead levels, the wrapper rather than the candy was tested. While the removal of candy from the children’s diet resulted in lowered blood lead levels, they remained well above 10 µg/dL suggesting the presence of other pathways of exposure. Focusing on Mexican candies shifts the blame to Mexican culture and individuals who choose to eat candies containing chili and tamarind while diverting attention away from lead hazards in the environment. 

#### 3.2.5. Water

Echoing the language of the SDWA, the California Health and Safety Codes require the use of lead-free pipes and fixtures in public water systems or in facilities where water is provided for human consumption (Sections 116875–116880). Similar to the SDWA “lead free” allows for 0.2% lead in solder and flux, 8% lead in pipes and pipe fittings, and 4% lead in plumbing fittings and fixtures [103]. California also requires the testing of consumers’ taps to assess lead and copper levels on an annual basis under the Lead and Copper Rule of the California Code of Regulations (Sections 64670–64690.80) [117]. It should be noted that under this policy water systems can transition testing consumers’ taps to every three years upon completing two consecutive sampling periods that result in lead and copper levels below actionable levels—0.005 µg/dL.

The Lead and Copper Rule leaves many questions unanswered, including the extent to which multi-family housing units are included in sampling frames. The rule prioritizes tier 1 sampling sites, which include single-family homes [117]. Multi-family housing units can be included in the sampling frame if they comprise more than 20% of a water system’s consumer base and/or there are not sufficient sing-family units to fulfill testing requirements. It should be noted that the rule requires water systems to test the water of any customer in the event lead is found to exceed permissible levels. However, the rule does not mandate the water system pay for these tests. Costs associated with testing water at the tap maybe a barrier for low-income households in requesting additional testing in their homes. Similar to the SDWA, “lead-free” plumbing under the California Health and Safety Codes contains lead. Hence, mirroring federal policies, California water safety policies still permit lead in water distribution systems. Permissible levels of lead, no matter how small, continue to put children, adults, and the elderly at risk for the detrimental health effects of lead [36]. 

#### 3.2.6. Testing

The Childhood Lead Poisoning Prevention Acts of 1986 and 1989 established California’s Childhood Lead Poisoning Prevention Program, and set guidelines for annual reporting of the prevalence, causes, and geographic occurrence of high blood lead levels among children. The Childhood Lead Poisoning Prevention Act of 1991 established screening protocols for lead testing. According to the act, all children considered “at risk” are to be tested for lead poisoning. The act defines “at risk” as children who spend a large portion of their day in a facility or building built prior to 1978; children who live near a former lead or steel smelter or industrial facility that historically or currently emits lead; children who live near a freeway or a high-traffic street; and children exposed to known sources of lead. The policy also established a fund to support the lead prevention program via fees levied against parties found responsible for lead contamination. The California Health and Safety Codes require testing laboratories to report the results of all blood lead tests they administer to the state electronically and include identifying information on the patient, the ordering physician, the analyzing laboratory, and the test performed (Section 124130); and stipulate that all health insurance plans provide comprehensive preventative care to children, including screening for blood lead levels for children of any age who are determined to be at risk by a medical provider (Section 1367.2). The California Insurance Codes mandate that all health insurances cover the cost of a blood lead test for children (Section 10118.8). Title 17 of the California Code Regulations mandate all children receiving public benefits be screened for lead poisoning at the ages of 12 and 24 months by a health care provider; outline the standard of care, including providing information on the health risks of lead to parents, testing all children who receive public benefits, and assessing the risk of lead poisoning among children not receiving public benefits; and require health care providers to intervene in the event that a child’s blood lead level exceeds 10 µg/dL and are expected to monitor the child until their blood lead level drops below 10 µg/dL [103].

Despite the presence of these policies, nearly three-fourths of children enrolled in Medi-Cal do not have their blood lead levels tested at the ages of 12 and 24 months [106], indicating that these policies do not adequately test and protect children that are at greatest risk for being lead poisoned, children of color and children who have a low-SES. Additionally, the water crisis in Flint, Michigan, points to the importance of preventing and testing for lead exposures among infants, with attention to lead exposure through formula feeding—an exposure pathway that has long been overlooked and made more vulnerable under conditions of socioeconomic inequities [3,7]. 

### 3.3. Lead Policies in Orange County, CA and the City of Santa Ana, CA 

Notably, we identified few lead-related policies at the local level. Lead policies enacted and/or implemented within the County are described in Table 4, below. 

#### 3.3.1. Lead Poisoning Prevention Education and Child Blood Lead Testing

The Orange County Childhood Lead Poisoning Prevention Program (CLPP) is implemented by the local Department of Public Health (Orange County Health Care Agency). The publicly stated goals of the Orange County CLPP include the provision of lead poisoning prevention education for caregivers, health care providers, and local agencies, and engaging in outreach to increase lead testing for children who are at heightened risk of lead exposure. Notably, online and in-person public communications through the local CLPP program acknowledge lead exposures through paint and soil, and emphasize lead exposures linked with candy and pottery from Mexico [118,119]. This communication strategy is rooted in a racialized individual behavior change (and blame) model and fails to emphasize the differential distribution of exposure to lead in the environment for communities of color and low-income communities. 

Minimization of population-based lead exposures—such as lead in paint, soil, and/or water—and an emphasis on potential lead exposures from foods and pottery from Mexico serves to heighten lead-related health inequities in two important ways. First, remediation of lead in paint, water, and soil falls within the purview of governmental agencies. Accordingly, lead exposures through these sources provide the greatest impact on population health and health equity. Given that low-income communities and communities of color are more likely to live in areas that are contaminated by lead [6,9,13], focusing communication strategies on lead in the built environment, and remediation options available to residents and communities, would disrupt the intergenerational cycle of exposure to legacy lead and exposure to ongoing sources of lead. Second, this messaging emphasizes lead exposures linked with consumer products from Mexico, which are alleged to be major sources of lead. However, as described above, the evidence base documenting lead in pottery and candy from Mexico is weak. As a consequence, such messaging serves to blame communities of color for lead exposures and to stigmatize engagement in practices that may affirm racial identities (e.g., frequenting ethnic grocery stores, obtaining and maintaining pottery from ancestral homelands). 

#### 3.3.2. School-Based Policies

Our policy review sought to identify two policy mechanisms by which lead prevention, mitigation, and/or remediation might unfold in school-based settings: (1) assessments of lead exposures in school settings (e.g., testing and addressing lead in paint, soil, and/or water at schools) and (2) supplemental educational services within school contexts that might become available to students identified as having been exposed to lead. 

Our review did not identify any lead-specific policies articulated by the Santa Ana Unified School District. Noticeably absent from the school district’s policies regarding individualized education programs [120], identification of individuals for special education [121], and discipline [122] are considerations of the influence of environmental exposures on student learning, educational performance, and behavior. Additionally, while academic assessments and in some cases health assessments inform the school district’s identification of children who may qualify for special education programs [121], Individuals with Disabilities Education Act programs [123], and/or transitional kindergarten [124], missing from stated policies are clinical assessments of current or past lead exposures and lead-related program eligibility. 

A well-established literature documents the association between early life lead exposures and adverse cognitive, behavioral, and academic outcomes [33,34,35,36,38] and carceral outcomes for youth and adults [43,44]. Additionally, evidence indicates links between school-based punishment and policing and incarceration [125]. Given this evidence base, notable gaps in Santa Ana Unified School District policies include: comprehensive assessments and remediation of lead exposures in the school environment (e.g., lead in water, paint, and/or soil); testing students for lead exposures; and strategic plans to ameliorate the social, educational, and health consequences of lead exposure. Evidence indicates that children in Santa Ana are 64% more likely than children across the State of California to have elevated blood lead levels [72,126]. Accordingly, this policy review points to the need for policies across the school district that are informed by the evidence indicating population-wide exposure to lead, with the goal of mitigating the cognitive, educational, and health impacts of lead exposure for students across the school district, rather than mitigating the academic and health consequences of lead exposure on a student-by-student basis. 

#### 3.3.3. Lead Remediation 

Once the health impacts of lead exposure have been documented for children, the Orange County Childhood Lead Poisoning Prevention Program, implemented through the Orange County Health Care Agency, provides case management services and identification of lead sources [119]. From public-facing documents, it remains unclear to the public whether the Orange County Health Care Agency provides support for remediation activities or ensures the protection of tenant rights if a residential lead exposure is identified [119]. 

Notably, our review did not identify any city-level policies that have been implemented to remediate lead. The paucity of local lead policies may be due in part to the deference of localities to federal and state policies. However, given the place-based nature of residential lead exposures, there is a missed opportunity to leverage local policies to remediate lead sources, in turn ameliorating lead exposures in residential settings over the life course and across generations. 

There are two local policy opportunities for which we are cautiously optimistic. First, a recent draft of the City of Santa Ana General Plan, which is actively under development, lists a potential policy pertaining to the remediation of health concerns such as lead-based paint within the strategic goal of enhancing and maintaining Santa Ana’s historic areas [127]. Second, in November 2018 a majority (70%) of residents approved the Measure I Bond for the Santa Ana Unified School District [128]. If implemented, this bond would provide funds to update classrooms and school infrastructure to improve student safety, including improving plumbing and electrical systems [128]. While this local policy landscape holds promise for remediating lead exposures in the built environment in Santa Ana residential, commercial, and educational settings, much vigilance is needed towards policy development and implementation, ensuring a focus on equity and lead exposures across the life course. 

## 4. Discussion

The purpose of this review is to examine the state of lead policies at the federal, state, and local levels, drawing upon the urban area of Santa Ana, CA as a case study to identify opportunities for intervention to promote health equity and prevent lead exposures. This multi-level lead policy review has three key findings. First, taken together, the patchwork of federal, state, and local lead policies tends to offer passive approaches to lead remediation and concentrate on lead-based paint in older housing stock. Similarly, policies and lead education programs offer limited attention to lead exposures from multiple sources (e.g., lead in paint, water, industrial emissions, and historical gasoline)—exposures that must be addressed from a population health and health equity perspective. Second, lead prevention efforts to date have largely focused on managing lead exposures. For example, rather than assessing – and addressing—lead in the environment, existing policies primarily treat children as an indicator of lead in the environment and in turn may implement mitigation or remediation activities after a designation of lead poisoning. Third, the endurance of lead exposures and inequities in lead exposures indicate a need for vigilance towards policy implementation and policy evaluation, with a focus on health equity. The following sections discuss limitations of lead policies to date and point to promising directions to prevent exposure to lead and reduce and eventually eliminate lead-related health inequities. We close with recommendations to reduce lead exposures, consider multiple lead exposure pathways, and reduce and eliminate health inequities related to lead.

### 4.1. Limitations of Lead Policies in the Twenty-First Century 

There are several limitations of these policies, which may contribute to persistent lead exposures for children, communities of color, and low-income communities. First, lead policies are segmented according to: source of lead, setting in which persons have been exposed to lead, and jurisdiction of the policy. This fragmentation of lead policies creates a crisis in which policies fail to address community-wide exposures and the cumulative and compounding effects of exposure to lead from multiple sources throughout the life course. Lead exposures do not occur in isolation or at one point in time. There are several reasons why lead exposures compound: lead exposures are encountered in multiple spheres of day-to-day life (e.g., workplace, residence, school, etc.); influence individuals, households, and broader communities; and can affect current and future health status. By focusing on individual households and dwellings, current lead policies miss an opportunity to address community-wide lead exposures. Relatedly, missing from these policies is a focus on lead exposures from current emission sources, such as e-waste recycling or industrial emissions, and historical lead (e.g., lead in gasoline, lead in former industries). Despite strong evidence of soil as an important exposure pathway, collectively these policies provide no guidelines for soil remediation beyond the presence of lead-based paint hazards. The fragmentation of lead-related policies, lack of support for remediation, and omission of compounding exposures frameworks contributes to the persistence of lead exposures and inequities in lead exposures. A multi-level and wholistic lens is important for addressing inequities, and is noticeably absent from current lead policies. 

Second, absent from lead policies is attention to ongoing and dynamic gentrification processes, which may exacerbate health inequities. For example, lower-income residents and persons of color are more likely to live in older housing that may have lead-based paint and/or have been exposed to historical emissions [6,15,16]. In addition to the displacement of lower-income residents and destabilization of housing markets [82], gentrification processes often involve the remodeling of homes. Remodeling may disturb lead on the property [6], in turn (re-)suspending lead in neighborhoods. Indeed, remediating lead paint has been demonstrated to increase lead exposures in a given household [6]. Thus, remodeling activities that accompany gentrification may amplify the effects of gentrification on health [129] by heightening longstanding residents’ exposure to lead as the neighborhood undergoes physical transformations to accommodate higher income residents. Lead remediation also contributes to lead exposures amongst construction workers involved in remediation activities [22,23], many of whom may be workers of color and/or immigrants [111]. Together, residents and construction workers may come to embody the hazardous environment that is being remediated and gentrified. Future work could examine the health equity implications of lead remediation in gentrifying neighborhoods, and lead exposures in the context of suburban poverty.

Third, despite evidence indicating that there is no safe level of lead exposure, child blood lead levels remain the criterion for activating any lead mitigation or remediation activities. Accordingly, inherent in these policies is the necessary demonstration of harm to child health, which can have lasting health consequences. Indeed, the threshold of lead exposure is high, and documentation of lead poisoning is necessary despite the reality that children are not routinely tested for lead exposure. Furthermore, FDA and HUD-designated “safe” lead exposure levels are linked with adverse health consequences [36]. Noticeably absent are policies regarding lead exposures for children older than 6 years of age, youth, adults, elders, and persons with chronic or infectious conditions that may be exacerbated by lead exposures. Moreover, stressful life conditions can exacerbate the health consequences of lead exposures. Greater attention is needed to occupational exposures for adults; the impact of lead exposures to all residents, through lead concentrations in air, soil, water, and dust; and lead exposure among children and other household members via pathways such as adults carrying dust back from occupational exposures. A deeper understanding and consideration of the cumulative impacts across multiple intersecting social statuses and identities, and of social, cognitive, and physical health consequences are needed. 

Fourth, this policy analysis suggests that the implementation of existing lead policies is relatively weak and passive, contributing to the endurance of lead exposures and lead-related inequities. For example, assessments of lead in the environment (e.g., residence, school, play areas) are not activated until a child has been diagnosed as having elevated blood lead levels. However, despite California’s policy mandate to test all children enrolled in Medi-Cal, an insufficient number of children are routinely tested for lead [106]. The inconsistent application of California’s testing policy prevents the activation of remediation activities. Additionally, policies that require an evaluation of lead exposures do not outline remediation options or funding mechanisms to support remediation activities. Given the disparate impact of lead exposures on communities of color and low-income communities, protections for workers, residents, and other members of affected communities need to be built into lead policies. A key implementation question underlying these lead policies pertains to how policies are enforced, and what happens when policies are violated. Notably, the EPA reports numerous civil penalties to responsible parties charged with not following lead mitigation and containment practices, many of which are reduced in amount once the EPA and the responsible party reach an agreement; with substantially fewer criminal charges filed. Findings from this review suggest a need to implement strategies and provide funding to safely remove lead from the environment and evaluate the equitable impact of lead policies. 

### 4.2. Recommendations for Health Equity Across the Life Course and Generations to Come

There are three key recommendations that emerged from our equity-oriented assessment of lead policies. First, to achieve equity, federal and state policies must adopt strategies to prevent individuals from being exposed to lead in the first place, alongside remediating hazardous environments. The legacies that lead leaves on the body indicate that current mitigating strategies (e.g., reducing exposure to chipping paint) and individual-level remediation strategies (e.g., remediating one house at a time) will not fully address the life course health consequences of lead exposures. For example, Rust and colleagues found that removing a portion of lead exposures in a household contributed to a 25% decrease in children’s blood lead levels [130]. These findings suggest that remediation activities are not sufficient in fully reducing children’s blood lead levels and that the deposition of lead in the bone may contribute to child blood lead levels. Moreover, childhood lead exposures affect health later in the life course: While the half-life of lead in blood is approximately one month [46,130], the half-life of lead in bones is 20 to 30 years [46,51,52]. Thus, to protect people at each stage of life, lead-related policies must adopt proactive strategies that prioritize human health and well-being over current or past industries that have contributed to lead in the environment and fully remediate settings contaminated with lead [131,132]. The cost of lead remediation is a commonly cited reason for the persistence of lead in the environment [132]. However, the social and health costs of lead exposures cannot be reduced to cost-saving frameworks such as those that guided decisions to include lead in consumer products in the first place. Recent litigation against paint companies for knowingly promoting and selling lead-based paint before it was officially banned [133] offer one such promising strategy available to localities for holding industries accountable and supporting community-level remediation activities. 

Second, policies are needed that address community-wide lead exposure pathways. In a study on the Bunker Hill Superfund site in Idaho, von Lindern and colleagues found that leaded dust in homes contributed 40% to 50% of children’s blood lead levels and approximately 60% of leaded dust in homes originates from community-wide soils, neighbors’ yards, and the home yard [134]. Remediating individual homes near the superfund site did lower children’s blood lead levels. However, the most effective strategy for reducing children’s blood lead levels proved to be remediation of community-wide soils including soils in surrounding properties and neighbors’ yards [134]. Thus, continuing to remediate one home at a time will do little to significantly reduce children’s blood lead levels because they will continue to be exposed to community-wide exposure pathways. To protect children, their families, and their community, community-wide lead hazards must be addressed. As long as federal and state policies continue to address one household or dwelling at a time, inequities in lead exposure will persist. 

Third, findings from this policy analysis point to a need to build an intergenerational, intersectional, and inter-sectoral movement to prevent and address lead exposures. The influence of lead exposures across multiple settings and from multiple sources suggest opportunities for bridging environmental, housing, educational, carceral, climate, economic, worker, and health justice initiatives. For example, as communities work to protect and create affordable housing, sustainable solutions lie in affordable, safe housing that is free of lead and other environmental toxins [135]. Likewise, as youth leaders in the (Anti-)Soil Lead Project have emphasized, educational equity and reform of the criminal justice system are linked with preventing lead exposures, and implementing policies and practices that are sensitive to the influence of past or current lead exposures on cognitive and educational outcomes. This may include, for example, school-district wide implementation of curriculum that is designed to interrupt the toll of lead exposures on cognitive outcomes and educational inequities; supporting a community-wide literacy network for children and youth of all ages; and supporting educational opportunities and holistic health initiatives. For example, youth in Santa Ana are actively organizing to disrupt what is experienced and characterized locally as school-to-prison-to-deportation pipelines [136]. Youth in Santa Ana are advocating for holistic approaches to youth development and school policies such as restorative justice at the school district level [136]. Likewise, communities may identify opportunities to partner with food sovereignty movements to provide access to healthy foods that can counterbalance the health effects of lead exposures [3]. For example, in Santa Ana, CA, youth are working to build and sustain a community garden and community supported agricultural system. Partnerships that identify and grow nutritious and affordable foods, ensure food is grown in safe environments, and connect gardeners with households who are disparately affected by lead exposures may provide a promising community-oriented approach to mitigating lead exposures. Additionally, occupational exposures to lead shape the health of workers and their household members in the short term and over the life course [20,22,23], and may be compounded by residential lead exposures. Accordingly, a holistic understanding of lead exposures provides an opportunity to bridge worker and environmental justice initiatives, particularly for workers in industrial and construction industries. Finally, climate change disparately affects low-income communities, communities of color, and older and younger community members [137,138]. Climate change also amplifies persistent and emerging environmental injustices [138]. With respect to lead in particular, fires, strong wind, and warmer water temperatures may re-suspend or agitate lead in the environment or water systems, exacerbating vulnerabilities for lead exposures. As such, it is critical to weave climate and environmental justice knowledge and action as communities adapt to, mitigate, and thrive in our changing climate. Health equity in all policies approaches may offer a unifying framework for evaluating intersecting and synergistic priorities across social movements, building a coalition, and developing multi-level change strategies that sustainably address priorities across justice initiatives. 

### 4.3. Strengths and Limitations

As with all analyses, this assessment has several limitations. First, the policies represented are not exhaustive. For example, federal policies included in this review do not include those implemented by the Food and Drug Administration (FDA) or Housing and Urban Development (HUD). Likewise, at the state and local level, environmental impact reports (EIRs) may include reporting of lead exposures or lead-related activities, though policies that mandate these reports, or government-related responses to the findings of EIRs were not included in this review. Future studies are warranted regarding the outcomes of EIRs, the extent to which communities were engaged in the process, and implications for health equity. Relatedly, our review did not include more granular policies that may affect particular workforces (e.g., teachers unions). Second, much of the established literature regarding lead exposures focuses on implications for children, and is quantitative in nature. Qualitative studies are needed that shed light on the complex experiences of living and/or working in communities affected by legacy or ongoing sources of lead; the social, educational, carceral, economic, and health impacts; and implications for individual, family, and community wellbeing across the life course. 

## 5. Conclusions

Findings suggest that current lead policies focus on individual sources of lead rather than cumulative or synergistic exposures, exposure settings, and populations exposed, with limited attention to policy implementation and health equity. Lead policies should incorporate a complex systems perspective, with a focus on cumulative exposures and health equity. Communities of color and low-income communities will continue to be affected disparately by health inequities linked with lead exposures unless we prevent lead exposures in the first place, and to fully remediate sources of lead.

## Figures and Tables

**Table 1:** Physical characteristics of the region and social and economic characteristics of the population, U.S., California, Orange County, CA, and Santa Ana, CA, 2013–2017.

Physical, Social, and Economic Characteristics	National (U.S.)	State (CA)	Local	
			Orange County, CA	Santa Ana, CA
**Percent of Residents Living in Census-Designated Urban Area** [75]	80.7%	95.0%	99.9%	100.0%
**Age of Housing Stock** [76]				
Built before 1980	54.7%	60.2%	61.6%	81.1%
Built after 1980	45.3%	39.8%	38.4%	18.9%
**Households below federal poverty level** [77]	**13.8%**	13.8%	10.7%	17.6%
**Age of Population** [78]				
<5 years	6.2%	6.4%	6.0%	7.6%
<18 years	22.9%	23.4%	22.7%	27.5%
18–34 years	23.4%	25.0%	23.9%	28.5%
35–64 years	38.8%	38.4%	39.8%	35.8%
65+ years	14.9%	13.2%	13.5%	8.1%
**Racial/Ethnic Identification** [79]				
Latina/o/x	17.6%	38.8%	34.2%	77.3%
Black, non-Latino	12.3%	5.5%	1.6%	0.8%
Asian, non-Latino	5.3%	13.9%	19.5%	11.4%
Other/Multi-racial, non-Latino	3.2%	3.9%	3.3%	1.1%
White, non-Latino	61.5%	37.9%	41.4%	9.4%
**Residents born outside of U.S.** [80]	13.4%	27.0%	30.3%	45.2%
**Residents with health insurance** [77]	89.5%	89.5%	89.6%	78.9%

*Source*: U.S. Census Bureau, American Community Survey 2017 5-Year Estimates from the following files: DP-02, DP-03, DP-04, DP-05, and B17017. 2010 American Community Survey estimates for percent of residents living in urban areas from file P2.

**Table 2:** Federal lead policies, 1976–present.

Policy	Setting		Source				
	Residential	Commercial	Air	Soil/Dust	Water	Paint	Consumer Products
Clean Air Act (1963)			✓				
Safe Drinking Water Act (1974)	✓	✓			✓		
Toxic Substances Control Act (1976)	✓	✓		✓	✓	✓	
Comprehensive Environmental Response, Compensation, and Liability Act (1980)		✓	✓	✓	✓		
Lead Contamination Control Act (1988)		✓			✓		✓
Residential Lead-Based Paint Hazard Act (1992)	✓			✓		✓	
Hazard Standards for Lead in Paint, Dust and Soil (TSCA Section 403) (2001)	✓	✓		✓		✓	
Lead Renovation, Repair and Painting Program Rules (2008)	✓	✓		✓		✓	
Consolidated Enforcement Response and Penalty Policy for the Pre-Renovation, Education Rule; Renovation, Repair and Painting Rule; and Lead-Based Paint Activities Rule (2010)	✓	✓					

**Table 3:** California lead policies.

Policy	Setting		Source				
	Residential	Commercial	Air	Soil/Dust	Water	Paint	Consumer Products
Childhood Lead Poisoning Prevention Acts (1986 and 1989)	✓			✓		✓	
Childhood Lead Poisoning Prevention Act (1991)	✓			✓		✓	
California Health and Safety Code Section 124130 (Laboratory Reporting)							
California Civil Code, Sections 1941.1; 1102–1102.16; California Health and Safety Code Sections 17961; 17980; 17920.10 (Housing)	✓			✓		✓	
California Health and Safety Code, Section 105250 (Training Accreditation)	✓	✓		✓		✓	
California Health and Safety Code, Section 1367.3 (Screening)							
California Insurance Code, Section 10119.8 (Screening)							
Lead-Safe Schools Protection Act (1992)		✓		✓	✓	✓	
California Health and Safety Code Sections 108550–108580 (Lead in Toys)							✓
California Health and Safety Code, Section 110552 (Lead in Candy)							✓
California Health and Safety Code, Sections 25214.1–25214.4.2 (Lead in Jewelry)							✓
California Health and Safety Code, Sections 116875–116880 (Lead in Plumbing)	✓	✓			✓		
California Health and Safety Code, Sections 105185–105197 (Occupational Lead Poisoning Prevention)							
California Code of Regulations, Sections 37000–37100 (Screening children enrolled in Medi-Cal)							
California Code of Regulations, Sections 35001 et seq (Accreditation and certification of workers)							
California Code of Regulations, Title 8, Sections 1532.1 et. Seq (Occupational lead exposure limits)							
California Code of Regulations, Sections 64670–64690.80 (Lead and Copper Rule)	✓	✓			✓		

**Table 4:** Orange County and Santa Ana, CA lead policies.

Policy or Government Program	Activities		
	Education	Referrals for Case Management	Home Investigation
Orange County Health Care Agency Childhood Lead Poisoning Prevention Program	✓	✓	✓
